# Utility of Targeted RNA Analysis in Neurogenetic Disorders

**DOI:** 10.1212/NXG.0000000000200341

**Published:** 2026-02-20

**Authors:** Shoji Ichikawa, Katie Yergert, Brooklynn Gasser, Meghan C. Towne, Dana Burow

**Affiliations:** From Ambry Genetics, Aliso Viejo, California

## Abstract

**Background and Objectives:**

Clinical genetic testing is a powerful diagnostic tool for neurologic disorders. However, its clinical utility is diminished by the large number of variants of unknown significance (VUS) detected in patients. While *in silico* predictive tools have improved, accurate classification of potentially spliceogenic variants still requires a demonstrated splicing impact. One major challenge for RNA studies for neurogenetic conditions is the access to disease-relevant tissues for analysis. In this study, we sought to determine how RNA studies of whole-blood RNA can effectively reclassify variants in genes associated with neurologic disorders.

**Methods:**

Thirty-eight potentially spliceogenic variants based on location and/or *in silico* predictions were identified in patients who underwent genetic testing (single gene, multigene panel, or exome) for a neurologic phenotype. These included 26 intronic (4 canonical and 22 noncanonical) and 12 exonic (8 missense, 2 nonsense, and 2 synonymous) variants. RNA isolated from the whole blood of the patients and healthy controls underwent targeted RT-PCR sequencing. Variants were assessed pre-RNA and post-RNA analysis based on the ACMG/AMP standards and guidelines. In addition, we reviewed the whole blood expression levels, based on the Genotype Tissue Expression (GTEx) Portal, for 212 genes commonly ordered for neurodevelopmental clinical genetic testing.

**Results:**

RNA analysis led to reclassification of 79% (30/38) of the variants. Eighteen variants became diagnostic (*i.e.*, VUS to pathogenic or likely pathogenic), and one likely pathogenic was upgraded to pathogenic. Eleven variants were downgraded to likely benign. The remaining variants did not change classification due to inconclusive RNA data (N = 3) or availability and/or strength of other lines of evidence (N = 5). Based on the GTEx expression data, 77% (164/212) of commonly ordered neurodevelopmental genes had expression in whole blood that is sufficient for targeted RNA analysis.

**Discussion:**

RNA studies provided molecular evidence enabling reclassification for the majority of variants. While diagnostic reclassifications provided a genetic answer for patients, downgrades also had an important impact by resolving VUS. Our data indicate that targeted RNA analysis using whole blood clarify the pathogenicity of potentially spliceogenic variants identified in clinical genetic testing and improve the accuracy of genetic diagnoses in neurologic disorders.

## Introduction

Clinical genetic testing is a powerful diagnostic tool for neurogenetic disorders. However, neurologic disorders are genetically heterogenous and often have nonspecific overlapping clinical features, including epilepsy, developmental delay, and intellectual disability. As a result, patients often undergo next-generation DNA sequencing–based genetic testing approaches using increasingly large multigene panels or clinical exome/genome sequencing. Exome/genome sequencing is also recommended as a first-tier genetic test for some neurodevelopmental indications.^1^ Together, these factors result in an inevitable increase in the number of variants being identified and reported, particularly on panel testing.^2^

Clinical laboratories are tasked with determining the clinical relevance of these variants using the current American College of Medical Genetics and Genomics and Association for Molecular Pathology (ACMG/AMP) standards and guidelines.^3^ Variants that may affect RNA splicing are particularly challenging to classify due to their unknown or variable effects on the transcript and consequently the protein function.

The canonical splice donor/acceptor sites within 2 nucleotides of the exon/intron boundary (±1, 2) have highly conserved dinucleotides, and disruption of these sites likely results in aberrant splicing. When loss of function is a known mechanism of disease, variants in the ±1, 2 position have a high prior probability of being pathogenic and can receive a very strong line of evidence (referred to as PVS1 in the ACMG/AMP guidelines).^3,4^ However, a recent study suggests that as many as 83% of rare spliceogenic variants may be located outside of the canonical splice sites.^5^ It remains challenging to interpret noncanonical variants, where the impact on splicing is more variable.

*In silico* predictive tools have improved over the years, and newer artificial intelligence–based splicing prediction tools, such as SpliceAI,^6^ Pangolin,^7^ and Spliceformer,^8^ provide increased confidence in the ability to identify variants with a potential splicing impact. Ultimately, variants outside of the ±1, 2 position are more likely to require experimental evidence to validate the predicted RNA impact. Without experimental evidence, noncanonical intronic variants and exonic variants with a potential splicing impact are typically classified as variants of uncertain significance (VUS).^9^ As a result, these VUS diminish the diagnostic yield of clinical genetic testing.

RNA splicing analysis targeting variants of interest can be used not only to detect aberrant splicing but also to quantify relative abundance of abnormal transcripts. While RNA studies using whole blood have proven successful in clarifying VUS in oncology diagnostics,^10,11^ their applicability in neurogenetic disorders remains unexplored. In this study, we sought to determine how targeted RNA analysis can improve diagnostic yield for patients with potentially spliceogenic variants in genes associated with neurologic disorders.

## Methods

### Variant Selection and Classification

**Figure 1:**
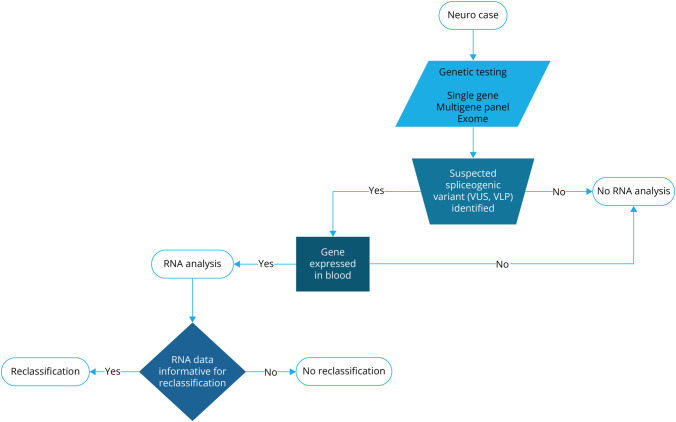
General Workflow of RNA Studies for Variants Detected in Clinical Genetic Testing Successful RNA analysis, which results in subsequent variant reclassification, requires thoughtful selection of variants. The impact of RNA evidence should be considered as variants with uncertain classifications (variant of uncertain significance [VUS] and variant, likely pathogenic [VLP]) are the most likely to benefit from additional evidence. Gene expression in whole blood is also crucial as blood is the most readily available tissue for clinical RNA studies. Therefore, suspected spliceogenic impact, variant classification before RNA analysis, and gene expression in blood tissue were considered in the triage process. Variants in genes with low expression in blood tissue were typically excluded from analysis.

Figure 1 illustrates the workflow from genetic testing to RNA analysis and classification in this study. Patients included in this study were found to have a potentially spliceogenic variant on clinical genetic testing for neurologic disorders at Ambry Genetics between 2014 and 2024—single gene (N = 4), multigene panel (N = 14), or exome sequencing (N = 16) (eTable 1). Qualifying variants were identified based on their predicted impact (using the best *in silico* tool available at the time of testing),^6,12-15^ location (near exon-intron boundary, generally within 5 nucleotides from an exon), expression in whole blood in Genotype-Tissue Expression (GTEx) Portal or bone marrow in the Human Protein Atlas,^16^ and/or potential for reclassification (current classification of VUS or likely pathogenic). A total of 38 variants from 34 patients were selected for targeted RNA analysis. Classification of the identified variants was determined pre-RNA and post-RNA analysis, using the current ACMG/AMP standards and guidelines for the interpretation of sequence variants^3^ in conjunction with an internally developed protocol available at the time of variant assessment (Ambry Genetics Variant Classification Scheme^17^). Where applicable, recent recommendations from the ClinGen SVI Splicing Subgroup were incorporated in the interpretation of RNA splicing evidence.^4^

### Targeted RNA Analysis

RNA analysis was performed based on laboratory practices at the time of testing and primarily done by massively parallel sequencing of RT-PCR products (CloneSeq or RT-PCRseq), as described previously.^18,19^ Importantly, all RNA studies included healthy controls that do not contain a reportable variant in the same gene and quantification of the transcripts to determine relative levels of aberrant splicing. In brief, whole blood was collected in PAXgene Blood RNA tubes from the patients with variant(s) of interest and healthy controls. Total RNA extracted from blood underwent reverse transcription and PCR amplification of the targeted region, followed by sequencing of the PCR product. Primers used in RT-PCR are available on request. For standard RT-PCR, PCR products were analyzed by capillary electrophoresis and cloned into plasmid vectors for transformation, followed by Sanger sequencing of DNA isolated from ∼20 individual colonies. For CloneSeq, PCR products were similarly cloned into plasmids, and a DNA library was generated from a pooled collection of transformed colonies to enable massively parallel sequencing. For RT-PCRseq, a DNA library was constructed directly from RT-PCR products for subsequent massively parallel sequencing. Sequencing reads were mapped to the hg19 human reference genome and analyzed using the proprietary Ambry bioinformatics pipeline. In both CloneSeq and RT-PCRseq workflows, the relative abundance of splicing events was quantified as percent spliced in (PSI), as previously described.^20^

### Gene Expression in Blood

To quantify the potential impact of RNA studies in genes with established neurologic disease associations, we selected 212 genes commonly ordered for neurodevelopmental clinical genetic testing. We reviewed expression levels of these genes in whole blood based on the GTEx Portal.^21^ Sufficient expression was defined as ≥0.5 median transcript per million (TPM) based on assay-specific internal data.

### Standard Protocol Approvals, Registrations, and Patient Consents

Western Institutional Review Board determined the study to be exempt from the Office for Human Research Protections Regulations for the Protection of Human Subjects (45 CFR 46) under Category 4. Retrospective data analysis of deidentified data exempted the study from the requirement to receive consent from patients.

### Data Availability

Data relating to classified alterations identified during diagnostic testing are deposited into ClinVar.^22^ Reasonable requests for additional anonymized data not published within this article will be made available by request from qualified investigators.

## Results

**Figure 2:**
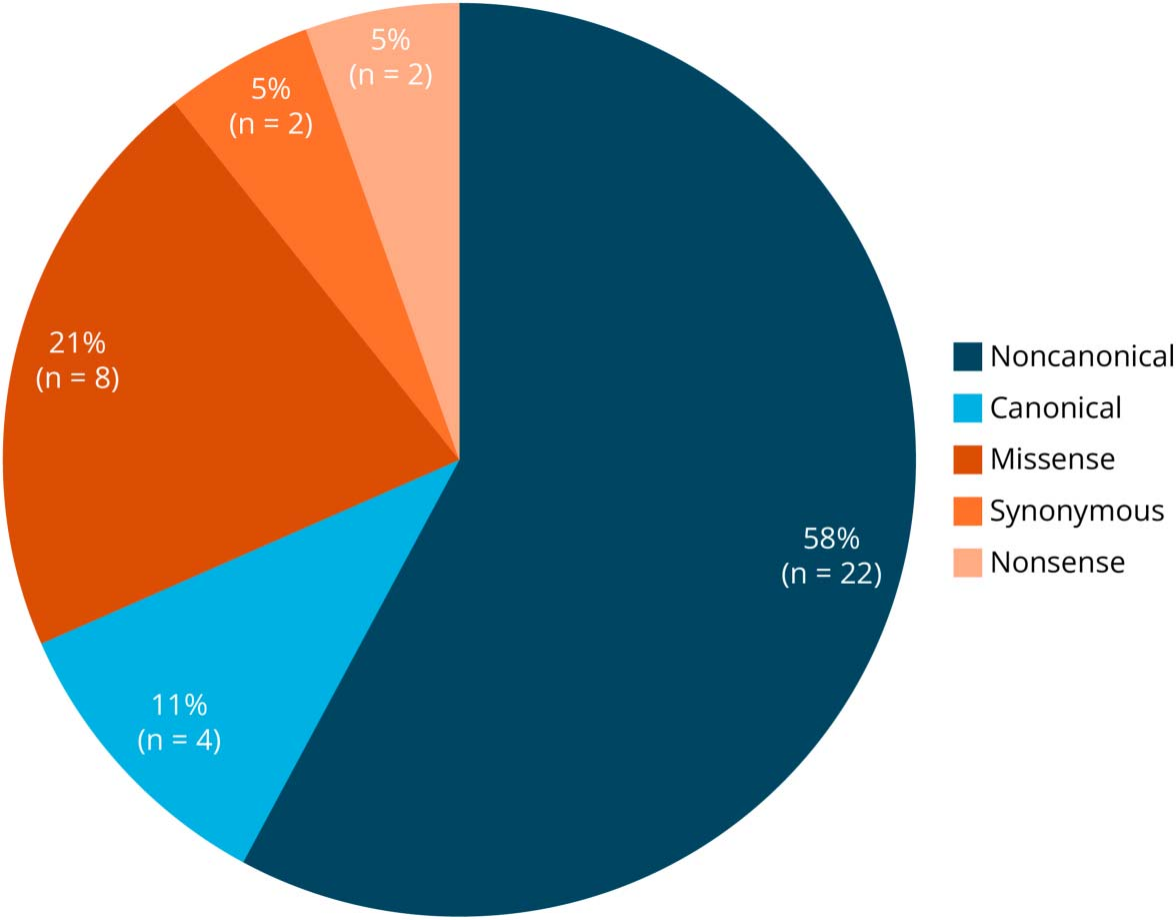
Percentage of Variant Types Included in RNA Analysis Intronic variant types are shown in shades of blue and exonic variant types are shown in shades of orange. Note that c.1531-9_1534dup13 was included in the intronic (non-canonical) group as the native donor site remains intact.

Demographic information of the patients included in this study is summarized in Table 1. Additional details on demographics, genotype, and phenotype for each patient are presented in eTable 1. Targeted RNA analysis assessed the splicing impact of 38 variants, including 34 single-nucleotide substitutions, 2 deletions, and 2 duplications (Table 2). Although 11% (4/38) of variants occurred at the canonical ±1, 2 position, most variants were located outside of the canonical position, including 58% (22/38) noncanonical intronic and 32% (12/38) exonic variants (Figure 2). Six missense variants affected either first or last nucleotide positions in an exon (Table 2). Most variants (95%, 36/38) were initially classified as VUS.

**Table 1:** Demographics of the 34 Patients in This Study

Sex	Number	Percent, (%)
Men	28	82
Women	6	18

R/E/A	Number	Percent, (%)
White	15	44
Multiple	5	15
Hispanic	3	9
Not provided	3	9
African American	2	6
Asian	2	6
Arabic	1	3
Ashkenazi Jewish	1	3
Bangladesh	1	3
Indian	1	3

Age at testing (y)	Number	Percent, (%)
0–5	12	35
6–10	12	35
11–15	4	11
16–20	3	9
21–30	1	3
31–40	2	6

Demographic and clinical information, including sex, race/ethnicity/ancestry (R/E/A), and age at testing, were collected from the test requisition form and supporting clinical documents provided by the ordering clinician.

**Table 2:** Classification of Variants Analyzed by Targeted RNA Analysis

Gene	Variant c. (p.)	SpliceAI	Classification^a^	ACMG/AMP codes^a^
*ADNP*	c.201G>C^b^ (p.Q67H)	DL 0.24, DG 0.01	VUS > LP	PS2, PS3, PM2
*ANKRD11*	c.226G>A^b^ (p.E76K)	DL 0.78	VUS > LP	PS3, PM2
*ATP13A2*	c.841-4A>G	AL 0.02, AG 0.03	VUS > LB	BS3
*ATRX*	c.4957-3A>G	AL 0.18, AG 0.96	VUS > LP	PS2, PS3, PM2
*CASK*	c.1503+3A>G	—	VUS > LB	BS3
*CC2D1A*	c.1357-2A>C	AL 1.00, AG 0.67	VUS	PP3
*CC2D1A*	c.2609G>A (p.R870Q)	AG 0.02	VUS	BP4, (BS3)
*CHD7*	c.6776-4T>G	AG 0.02	VUS > LB	BS3
*COQ4*	c.202+4A>C	DL 0.01, DG 0.12	VUS	PM2, PM3
*CREBBP*	c.3251-3C>T	—	VUS > LB	BS2, BS3
*CUX1*	c.302-2A>C	AL 0.99, AG 0.41	VUS	PS3, PS4, PM2
*DDX3X*	c.284+3A>T	—	VUS > LB	BS3, BS2
*DEPDC5*	c.3155+5G>A	DL 0.55, DG 0.01	VUS > P	PS3, PM2
*FLNA*	c.1065+3G>A	—	VUS > LB	BS3
*FMR1*	c.104+3_104+6delAAGT	DL 1.00, DG 0.03	VUS > P	PS2, PS3, PM2
*FOXP1*	c.1531-9_1534dup13	AL 0.98, AG 0.99	VUS > P	PS2, PS3, PM2
*FOXP1*	c.1652+5G>C	DL 0.95, DG 0.34	VUS > P	PS2, PS3, PM2
*FRYL*	c.1987-1G>A	AL 0.94, AG 0.84	VUS > P	PS2, PS4, PS3, PM2
*HNRNPK*	c.516+2dupT	DL 0.21, DG 0.46	VUS	PM2, PP3
*HUWE1*	c.46-5C>G	AL 0.01, AG 0.11	VUS > LB	BS3
*NF1*	c.586G>A^b^ (p.E196K)	DL 0.05	VUS > LP	PS3, PM2
*NF1*	c.6280C>A (p.P2094T)	DG 0.90	VUS > LP	PS3, PM2
*NF1*	c.6757G>T^c^ (p.A2253S)	AL 0.30	VUS > P	PS3, PS4, PM2, PM6
*OPHN1*	c.1224C>A (p.Y408*)	AL 0.34	LP > P	PVS1, PS4, PM2, (BS3)
*PRICKLE1*	c.435G>A (p.A145A)	AL 0.02, AG 0.14	VUS > LB	BS3
*PTCH1*	c.3549+1G>A	DL 0.89, DG 0.01	LP	PS3, PS4, PM2
*PTEN*	c.210-12C>G	AL 0.67, AG 0.79	VUS > LP	PS2, PS3, PM2
*SETD5*	c.3301C>T (p.Q1101*)	DG 0.99	VUS > P	PVS1, PS2, PM2
*SPG11*	c.5866+5G>C	DL 0.72	VUS > LP	PS3, PM2, PM3
*SYNJ1*	c.968G>A^b^ (p.R323K)	—	VUS	BS3
*SZT2*	c.2472-5T>C	AL 0.01, AG 0.06	VUS > LB	BS3
*TNNT1*	c.32+5G>A	DL 0.94	VUS > LP	PS3, PM2
*TRIP12*	c.4190+5G>A	DL 0.78, DG 0.29	VUS > P	PS2, PS3, PM2
*TSC1*	c.2041G>A^b^ (p.G681S)	DL 0.62	VUS > P	PS3, PS4, PM2
*TSC2*	c.3918C>T (p.G1306G)	DG 0.02	VUS > LB	BS3
*VPS13B*	c.2516-3T>A	AL 0.46	VUS > LB	BS3
*WDR45*	c.976+5_976+10delGTGGGA	DL 0.41, DG 0.07	VUS > LP	PS2, PS3, PM2
*WHSC1*	c.2881+5G>A	DL 0.24, DG 0.03	VUS	PS3, PS4, PM2

Abbreviations: AG = acceptor gain; AL = acceptor loss; B = benign; DG = donor gain; DL = donor loss; - = no predicted impact; LB = likely benign; LP = likely pathogenic; P = pathogenic; VUS = variant of uncertain significance.

a Classifications (pre-RNA and post-RNA analysis) and codes from the ACMG/AMP guidelines^3^ are based on the information available at the time of reassessment following RNA analysis and their application may differ from current practice. Where RNA data provided evidence, PS3 and BS3 were applied instead of PP3 and BP4, respectively. (BS3) denotes that a variant had little or no splicing impact in RNA analysis, but the code was not applied in the final classification (see texts for details).

b Last nucleotide of an exon.

c First nucleotide of an exon.

The *in silico* predictor, SpliceAI, projected that 24/38 of the variants selected for this study would result in aberrant splicing (SpliceAI score ≥0.2, moderate level of evidence for spliceogenicity^4^), of which 21 (88%) variants were confirmed to have a demonstrated splicing impact (eTable 2). Of the 3 remaining variants, 2 had inconclusive RNA results. *VPS13B* c.2516-3T>A had no apparent splicing impact, despite a relatively high SpliceAI score (acceptor loss, 0.46), and it was downgraded to likely benign.

**Figure 3:**
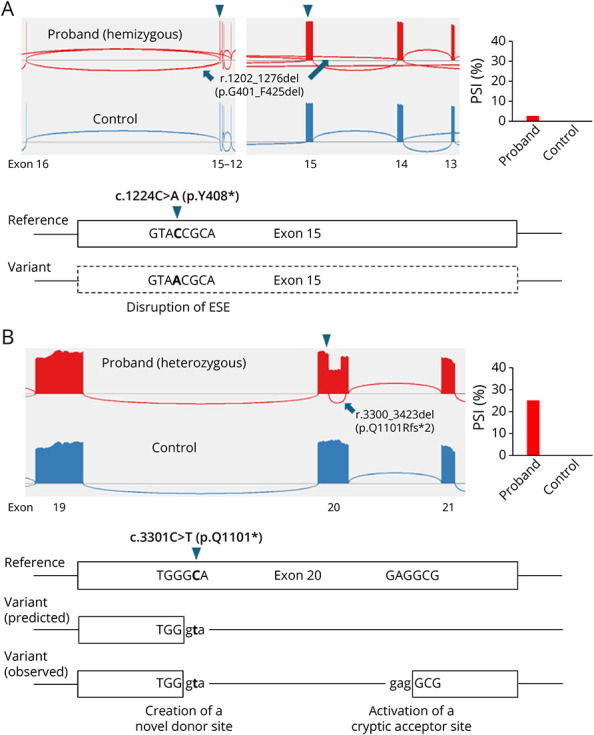
Targeted RNA Analysis of 2 Spliceogenic Nonsense Variants Targeted RT-PCRseq was performed on blood RNA from the proband with a nonsense variant and variant-negative healthy control: (A) *OPHN1* c.1224C>A (p.Y408*) and (B) *SETD5* c.3301C>T (p.Q1101*). (Top) Sashimi plot of RT-PCRseq and quantification of the RT-PCRseq results shown as percent spliced in (PSI). (Bottom) Schematics of the splicing impact. Triangles indicate the location of the variant. (A) The *OPHN1* variant was predicted to affect the adjacent acceptor site (SpliceAI acceptor loss, 0.34), leading to skipping of exon 15 (r.1202_1276del, p.G401_F425del). Although this variant does not have a direct effect on the acceptor site, it is predicted to disrupt one of the potential exonic splicing enhancers (ESE) recognized by the splicing factor SC35 (reference 2.78365, variant 0.08551) based on the *in silico* analysis using ESEfinder.^14^ Weakening of this ESE likely contributed to the exon skipping (dashed box), albeit low level, in RNA analysis. Of note, skipping of exons 13–15 (r.1105_1276del, p.Y370Lfs*23) was detected at PSI 2.97% in the proband but was absent in the control. This splicing event was excluded in the PSI figure as its association with the variant is unclear at this time. (B) The *SETD5* variant was predicted to create a novel donor site (SpliceAI donor gain, 0.99), leading to partial exon skipping (r.3300_3497del, p.Q1101_W1166del). RNA analysis showed that the variant not only created the predicted donor site but also activated a nearby cryptic acceptor site within exon 20, resulting in out-of-frame transcript (r.3300_3423del, p.Q1101Rfs*2).

In most cases (18/21), the confirmation of aberrant splicing enabled the variant classification to be upgraded. In 2 cases where aberrant splicing might rescue the impact of a nonsense variant, RNA analysis played a crucial role for upgrading variant classification. In the case of *OPHN1* c.1224C>A (p.Y408*), SpliceAI predicted the loss of the adjacent native splice acceptor site, which would lead to a small in-frame deletion (p.G401_F425del) and rescue the nonsense variant from nonsense-mediated decay (NMD)—*i.e.*, deletion of the nonsense codon (Figure 3A). Given this potential outcome, the PVS1 weight was reduced to strong in the initial classification, and the variant was classified as likely pathogenic. Although RNA analysis detected the in-frame deletion transcript, it accounted for <3% of total RNA product and deemed insufficient to rescue the effect of the nonsense (Figure 3A, eTable 2). In this case, the lack of aberrant RNA splicing was used to inform the most likely mutational mechanism of this variant, allowing us to confidently apply full PVS1 weight and upgrade the variant to pathogenic (Table 2). In the other case, *SETD5* c.3301C > T (p.Q1101*) was predicted to create a novel donor site, leading to an in-frame deletion (p.Q1101_W1166del) (Figure 3B). This deletion also had the potential of rescuing the effect of the nonsense codon. RNA analysis revealed that it has a splicing impact; however, the observed transcripts were different from the prediction. The major abnormal transcript, accounting for about 25% of the total transcripts, was out-of-frame (p.Q1101Rfs*2), and <2% of the transcripts were an in-frame deletion (p.Q1101_R1157del) not predicted by SpliceAI (Figure 3B, eTable 2). As a result, this variant was expected to trigger NMD due to the presence of a (different) premature termination codon introduced by the nonsense variant and out-of-frame transcript. This information allowed us to apply PVS1 at full strength and upgrade the variant from VUS to pathogenic.

By contrast, SpliceAI predicted that 14/38 of the variants selected for this study would not or were less likely to result in aberrant splicing (SpliceAI score <0.2), of which 12 (86%) variants were confirmed to have no demonstrated splicing impact (eTable 2). The results allowed us to downgrade 10 of the 12 variants. However, the remaining 2 variants had inconclusive RNA results (*COQ4* c.202+4A>C) or demonstrated a splicing impact (*NF1* c.586G>A). SpliceAI missed the splicing impact of the *NF1* variant, which affects the last nucleotide of exon 5 (donor loss, 0.05). RNA analysis revealed skipping of exon 5 (r.480_586del) in both the proband and their parent carrying the same variant and was absent in the control sample (eFigure 1). The result allowed us to reclassify this variant from VUS to likely pathogenic.

**Figure 4:**
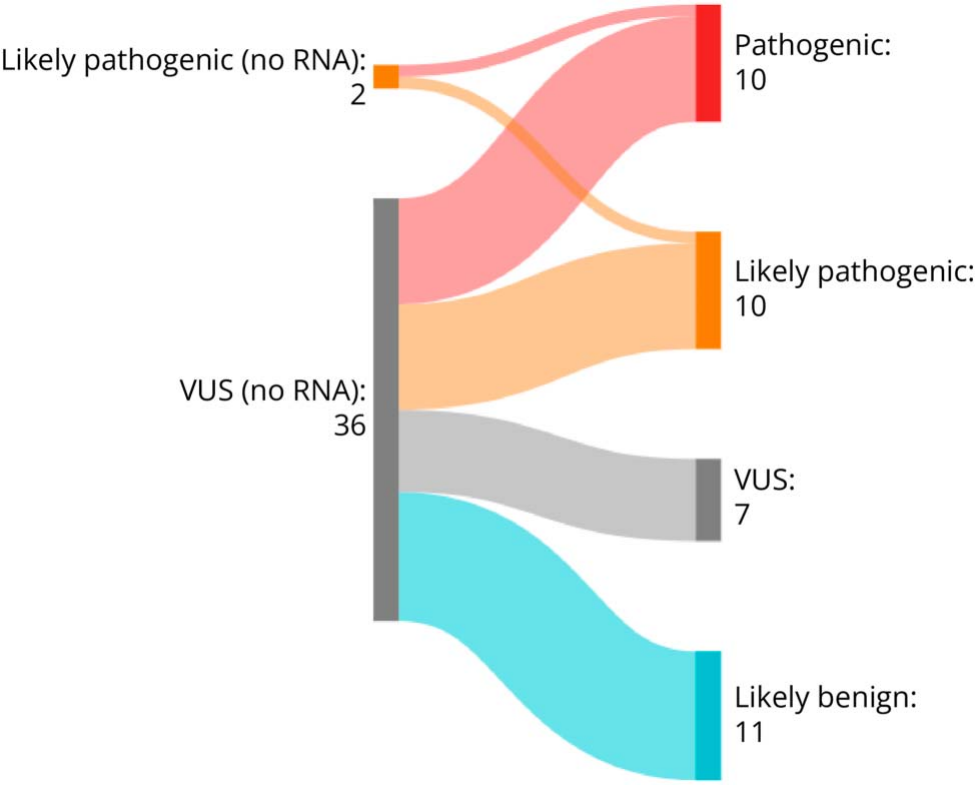
Variant Classifications Pre- and Post-RNA Analysis Comparison of variant classification before RNA analysis (left side of plot) and post RNA analysis (right side of plot). A total of 38 variants were tested, and RNA evidence supported the reclassification of 30 variants (79%).

In total, retrospective RNA analysis aided in the reclassification of 79% (30/38) of the variants evaluated (Figure 4, Table 2). Eighteen VUS became diagnostic (*i.e.*, VUS to pathogenic or likely pathogenic), and one likely pathogenic was upgraded to pathogenic. In addition, 11 variants were downgraded to likely benign based on the RNA evidence. Eight variants did not change classification after RNA analysis, but the analysis still provided useful information for 3 of those variants. Two missense VUS were considered potentially spliceogenic. *CC2D1A* c.2609G>A (p.R870Q) had a predicted splicing impact, albeit weak, and *SYNJ1* c.968G>A (p.R323K) was located at the last nucleotide of an exon. Both had no aberrant splicing detected in RNA studies, and we were able to rule out their splicing impact. However, these variants could not be downgraded due to their unknown impact at the protein level. One canonical variant, *PTCH1* c.3549+1G>A, was classified as likely pathogenic at the initial assessment and RNA analysis demonstrated a splicing impact as predicted; however, it could not be upgraded due to an atypical clinical presentation in the patient. Therefore, only 13% (5/38) variants had either inconclusive or insufficient RNA data, and RNA studies provided new information on 87% (33/38) of the variants tested in this study.

This study was limited to 34 neurologic genes, but based on gene expression data from the GTEx project, 77% (164/212) of the neurodevelopmental genes screened were above the cutoff for sufficient expression in whole blood (eTable 3). Thus, a variant in these genes would likely benefit from RNA analysis if an appropriate variant were detected as part of clinical genetic testing.

## Discussion

Our study used targeted RNA analysis to detect and quantify aberrant splicing in 34 different genes associated with neurologic disorders. In total, 79% (30/38) of the variants tested were reclassified, and 53% (18/34) of the patients received a genetic diagnosis, indicating that RNA analysis is a powerful tool for variant classification that improves diagnostic yield for patients with neurogenetic diseases. The relatively high reclassification rate may reflect our selection of variants as the study included variants with sufficient gene expression in blood and high likelihood of being pathogenic or benign based on *in silico* prediction. This study also highlights the limitation of *in silico* prediction. While *in silico* tools are useful for variant classification, we observed discrepancies between *in silico* predictions and experimental data for 13% (5/38) of the variants, supporting the necessity of RNA analysis for the most accurate classification. For example, *NF1* c.586G>A, which affects last nucleotide position, had a low predicted impact (donor loss, 0.05), but RNA analysis revealed that it has a substantial splicing impact. By contrast, *VPS13B* c.2516-3T>A did not show aberrant splicing in RNA studies, despite a moderate predicted impact (acceptor loss, 0.46). These discrepancies emphasize why direct RNA evidence is critical to accurate variant classification and patient diagnoses for rare diseases.

For accurate variant classification, interpretation of RNA evidence must consider the quantity of the observed transcripts as well as predicted molecular consequences at the RNA and protein levels. Abnormal transcripts detected by RNA analysis can have different impacts on the reading frame. Out-of-frame transcripts introducing a premature termination codon (upstream of 50–55 nucleotides of penultimate exon) are predicted to undergo NMD and thus considered deleterious. By contrast, abnormal splicing events preserving the reading frame (for example, in-frame exon skipping) may be tolerated if the affected position/region is not critical to the protein function. In addition to identifying aberrant splicing, we demonstrated that quantification of abnormal transcripts is a crucial insight from RNA analysis. As illustrated by the 2 nonsense variants (*OPHN1* c.1224C>A and *SETD5* c.3301C>T), the magnitude of aberrant splicing can have a major impact on the interpretation of RNA data and subsequent variant classification.

It is important to note that RNA analysis does not always result in variant reclassification, as observed in 21% (8/38) of the variants in this study. The strength of experimental RNA evidence can be insufficient due to variable quality and strength of RNA data. RNA data were inconclusive for 3 variants. One such variant was *CC2D1A* c.1357-2A>C, which was predicted to have a splicing impact (acceptor loss 1.00; acceptor gain 0.67). It occurs at a NAGNAG site—a tandem acceptor site where either NAG motif can function as a 3′ splice site and yield transcript isoforms differing by 3 nucleotides (*i.e.*, in-frame deletion/addition of 1 amino acid). NAGNAG sites are evolutionarily conserved and estimated to occur in 30% of human genes.^23^ However, the functional consequences of a subtle change in protein structure may be insufficient to cause disease. As a result, we were unable to assign any evidence to the RNA data. There were also 2 missense variants that had no splicing impact in RNA studies, but these variants remained VUS due to their unknown impact at the protein level. In the setting of a nonspecific phenotype, demonstration of aberrant splicing may not be sufficient to change variant classification. Our RNA analysis of the variant *PTCH1* c.3549+1G>A showed the expected skipping of the adjacent exon. This variant was detected in a 2-year-old child with developmental delay and macrocephaly (eTable 1). Notably, the patient did not fulfill the major diagnostic criteria of *PTCH1-*associated nevoid basal carcinoma syndrome. The patient's parent exhibited a history of postaxial polydactyly and a single basal cell carcinoma, while siblings presented with polydactyly, pectus carinatum, or cardiac fibromas. Although the clinical features within this family were highly suggestive, none met the major criteria for *PTCH1*-associated nevoid basal carcinoma syndrome. As a result, the variant remained classified as likely pathogenic. As highlighted by these examples, even if RNA analysis provides evidence toward the pathogenicity or benignity, some variants cannot be reclassified since the final classification depends on the availability and/or strength of other lines of evidence.^3^

There are several limitations in this study. First, most genes are expressed in specific tissue or cell types, and expression/splicing is regulated differentially at developmental stages.^24^ The lack of access to disease-relevant tissues is the major challenge for any RNA analysis. For many neurogenetic disorders, nervous tissue may be ideal, but it is not readily available. Blood or skin fibroblasts must be used as a surrogate. Splicing events observed in blood may not accurately reflect those occurring in nervous tissues. Nonetheless, the risk of misclassification is likely low as other lines of evidence are included in variant assessment. Furthermore, even in the scenarios where we do not apply clinical evidence such as PS2, PS4, and BS2 (Table 2), a phenotype overlap or its absence is always considered to ensure accurate classification. Second, severe neurogenetic disorders are rare, and variants of interest are often not found in any other individuals. While our RNA studies included variant-negative healthy controls to confirm the observed splicing is specific to the variant of interest, lack of samples from other individuals with the same variant limits our ability to assess variability between individuals. Third, our RNA assay and variant classification were not uniform across all variants. While quantification of aberrant splicing has been of the utmost importance in all our RNA assays, the initial assay design was less quantitative and sequenced a limited number of cloned RT-PCR products. Variant classification is also not static. The interpretation and weight of RNA evidence is continuously improved as we learn nuances of RNA data and incorporate new ClinGen SVI recommendations.^4,25,26^ Finally, there is a limit on the size of transcripts that can be amplified by routine targeted RT-PCR. As a result, the assay may not be able to detect large insertions such as full intron retention. This inability to detect aberrant splicing is particularly concerning for downgrades. However, it should be noted that almost all downgrades in this study had *in silico* predictions supporting benignity and they were not downgraded based on the RNA evidence alone.

Our study showed that RNA analysis can provide essential information for variant classification in neurogenetic disorders. As mentioned earlier, one limitation for RNA analysis in neurologic genes is the lack of access to nervous tissues and need to test alternative tissue types, namely, blood, which may or may not express the gene of interest. In fact, before establishing the current workflow (Figure 1), we tested additional variants not included in this study, and we were unable to complete RNA studies for some genes (for example, *TBR1*, *CACNA1A*, *FOLR1*, and *PLP1*), which have minimal to no expression in whole blood. Our examination of more than 200 neurologic genes in the GTEx Portal showed that 77% have sufficient expression in blood (median TPM ≥0.5). A previous study reported that 76% of 284 neurologic genes had median TPM ≥0.1 in blood.^27^ These observations highlight the high potential for blood RNA studies in neurogenetic diseases. It is worth noting that the gene expression threshold (median TPM ≥0.5) is only used as a general screening metric. One of the genes included in this study, *OPHN1*, had 0.0973 TPM, but RNA studies were successfully performed. This suggests that a higher percentage of the genes may be appropriate for RNA studies.

In summary, targeted RNA analysis using RNA from whole blood provided molecular evidence supporting pathogenicity or benignity for the vast majority of variants and resulted in the reclassification of 79% of the variants. Half of the variants included in this study had diagnostic reclassifications. Equally important were the downgrades of the other variants that would have remained VUS without RNA analysis. Although RNA-based studies are limited to genes expressed in the blood or other readily obtainable tissues, our data indicate that RNA analysis can be used to clarify the pathogenicity of potential spliceogenic variants and improves the accuracy of genetic diagnosis of neurologic disorders. As the field of medical genetics shifts from exome sequencing to genome sequencing as the first-line diagnostic approach, an increasing number of potential spliceogenic variants is being identified within intronic regions that are not typically interrogated by multi-gene panel testing or exome sequencing. Consequently, RNA analysis, as delineated in this study, is anticipated to assume an even greater significance in diagnosis of neurogenetic disorders.

## Glossary

GTEx: Genotype Tissue Expression
TPM: transcript per million

## References

[1] Manickam K, McClain MR, Demmer LA, et al.; ACMG Board of Directors. Exome and genome sequencing for pediatric patients with congenital anomalies or intellectual disability: an evidence-based clinical guideline of the American College of medical genetics and Genomics (ACMG). Genet Med. 2021;23(11):2029-2037. doi:10.1038/s41436-021-01242-6 34211152

[2] Rehm HL, Alaimo JT, Aradhya S, et al., Medical Genome Initiative Steering Committee. The landscape of reported VUS in multi-gene panel and genomic testing: time for a change. Genet Med. 2023;25(12):100947. doi:10.1016/j.gim.2023.100947 37534744 PMC10825061

[3] Richards S, Aziz N, Bale S, et al.; ACMG Laboratory Quality Assurance Committee. Standards and guidelines for the interpretation of sequence variants: a joint consensus recommendation of the American College of medical genetics and Genomics and the association for molecular Pathology. Genet Med. 2015;17(5):405-424. doi:10.1038/gim.2015.30 25741868 PMC4544753

[4] Walker LC, Hoya Mdl, Wiggins GAR, et al.; ClinGen Sequence Variant Interpretation Working Group. Using the ACMG/AMP framework to capture evidence related to predicted and observed impact on splicing: recommendations from the ClinGen SVI splicing Subgroup. Am J Hum Genet. 2023;110(7):1046-1067. doi:10.1016/j.ajhg.2023.06.002 37352859 PMC10357475

[5] Chong R, Insigne KD, Yao D, et al. A multiplexed assay for exon recognition reveals that an unappreciated fraction of rare genetic variants cause large-effect splicing disruptions. Mol Cell. 2019;73(1):183-194.e8. doi:10.1016/j.molcel.2018.10.037 30503770 PMC6599603

[6] Jaganathan K, Kyriazopoulou Panagiotopoulou S, McRae JF, et al. Predicting splicing from primary sequence with deep learning. Cell. 2019;176(3):535-548.e24. doi:10.1016/j.cell.2018.12.015 30661751

[7] Zeng T, Li YI. Predicting RNA splicing from DNA sequence using Pangolin. Genome Biol. 2022;23(1):103. doi:10.1186/s13059-022-02664-4 35449021 PMC9022248

[8] Jónsson BA, Halldórsson GH, Árdal S, et al. Transformers significantly improve splice site prediction. Commun Biol. 2024;7(1):1616. doi:10.1038/s42003-024-07298-9 39633146 PMC11618611

[9] Truty R, Ouyang K, Rojahn S, et al. Spectrum of splicing variants in disease genes and the ability of RNA analysis to reduce uncertainty in clinical interpretation. Am J Hum Genet. 2021;108(4):696-708. doi:10.1016/j.ajhg.2021.03.006 33743207 PMC8059334

[10] Horton C, Hoang L, Zimmermann H, et al. Diagnostic outcomes of concurrent DNA and RNA sequencing in individuals undergoing hereditary cancer testing. JAMA Oncol. 2024;10(2):212-219. doi:10.1001/jamaoncol.2023.5586 37924330 PMC10625669

[11] Karam R, Conner B, LaDuca H, et al. Assessment of diagnostic outcomes of RNA genetic testing for hereditary cancer. JAMA Netw Open. 2019;2(10):e1913900. doi:10.1001/jamanetworkopen.2019.13900 31642931 PMC6820040

[12] Desmet F-O, Hamroun D, Lalande M, Collod-Béroud G, Claustres M, Béroud C. Human splicing finder: an online bioinformatics tool to predict splicing signals. Nucleic Acids Res. 2009;37(9):e67. doi:10.1093/nar/gkp215 19339519 PMC2685110

[13] Yeo G, Burge CB. Maximum entropy modeling of short sequence motifs with applications to RNA splicing signals. J Comput Biol. 2004;11(2-3):377-394. doi:10.1089/1066527041410418 15285897

[14] Cartegni L, Wang J, Zhu Z, Zhang MQ, Krainer AR. ESEfinder: a web resource to identify exonic splicing enhancers. Nucleic Acids Res. 2003;31(13):3568-3571. doi:10.1093/nar/gkg616 12824367 PMC169022

[15] Reese MG, Eeckman FH, Kulp D, Haussler D. Improved splice site detection in genie. J Comput Biol. 1997;4(3):311-323. doi:10.1089/cmb.1997.4.311 9278062

[16] Uhlén M, Fagerberg L, Hallström BM, et al. Proteomics. Tissue-based map of the human proteome. Science. 2015;347(6220):1260419. doi:10.1126/science.1260419 25613900

[17] Ambry Genetics® Variant Classification Scheme. ambrygen.com/science/variant-classification

[18] Horton C, Cass A, Conner BR, et al. Mutational and splicing landscape in a cohort of 43,000 patients tested for hereditary cancer. NPJ Genom Med. 2022;7(1):49. doi:10.1038/s41525-022-00323-y 36008414 PMC9411123

[19] Farber-Katz S, Hsuan V, Wu S, et al. Quantitative analysis of BRCA1 and BRCA2 germline splicing variants using a novel RNA-massively parallel sequencing assay. Front Oncol. 2018;8:286. doi:10.3389/fonc.2018.00286 30101128 PMC6072868

[20] Schafer S, Miao K, Benson CC, Heinig M, Cook SA, Hubner N. Alternative splicing signatures in RNA-seq data: percent spliced in (PSI). Curr Protoc Hum Genet. 2015;87:11.16.1-11.16.14. doi:10.1002/0471142905.hg1116s87 26439713

[21] GTEx Portal. Accessed November 01, 2023. gtexportal.org/home/

[22] ClinVar. ncbi.nlm.nih.gov/clinvar/

[23] Hiller M, Huse K, Szafranski K, et al. Widespread occurrence of alternative splicing at NAGNAG acceptors contributes to proteome plasticity. Nat Genet. 2004;36(12):1255-1257. doi:10.1038/ng1469 15516930

[24] Kukurba KR, Montgomery SB. RNA sequencing and analysis. Cold Spring Harb Protoc. 2015;2015(11):951-969. doi:10.1101/pdb.top084970 25870306 PMC4863231

[25] Brnich SE, Abou Tayoun AN, Couch FJ, et al.; Clinical Genome Resource Sequence Variant Interpretation Working Group. Recommendations for application of the functional evidence PS3/BS3 criterion using the ACMG/AMP sequence variant interpretation framework. Genome Med. 2019;12(1):3. doi:10.1186/s13073-019-0690-2 31892348 PMC6938631

[26] Abou Tayoun AN, Pesaran T, DiStefano MT, et al.; ClinGen Sequence Variant Interpretation Working Group ClinGen SVI. Recommendations for interpreting the loss of function PVS1 ACMG/AMP variant criterion. Hum Mutat. 2018;39(11):1517-1524. doi:10.1002/humu.23626 30192042 PMC6185798

[27] Frésard L, Smail C, Ferraro NM, et al. Identification of rare-disease genes using blood transcriptome sequencing and large control cohorts. Nat Med. 2019;25(6):911-919. doi:10.1038/s41591-019-0457-8 31160820 PMC6634302

